# Disease-dependent interaction policies to support health and economic outcomes during the COVID-19 epidemic

**DOI:** 10.1101/2020.08.24.20180752

**Published:** 2020-09-01

**Authors:** Guanlin Li, Shashwat Shivam, Michael E. Hochberg, Yorai Wardi, Joshua S. Weitz

**Affiliations:** 1 Interdisciplinary Graduate Program in Quantitative Biosciences, Georgia Institute of Technology, Atlanta, GA, USA.; 2 School of Physics, Georgia Institute of Technology, Atlanta, GA, USA.; 3 School of Electrical and Computer Engineering, Georgia Institute of Technology, Atlanta, GA, USA.; 4 ISEM, Université de Montpellier, CNRS, IRD, EPHE, Montpellier, France.; 5 Santa Fe Institute, Santa Fe, NM 87501, USA.; 6 School of Biological Sciences, Georgia Institute of Technology, Atlanta, GA, USA.

## Abstract

Lockdowns and stay-at-home orders have partially mitigated the spread of Covid-19. However, the indiscriminate nature of mitigation — applying to all individuals irrespective of disease status — has come with substantial socioeconomic costs. Here, we explore how to leverage the increasing reliability and scale of both molecular and serological tests to balance transmission risks with economic costs involved in responding to Covid-19 epidemics. First, we introduce an optimal control approach that identifies personalized interaction rates according to an individual’s test status; such that infected individuals isolate, recovered individuals can elevate their interactions, and activity of susceptible individuals varies over time. Critically, the extent to which susceptible individuals can return to work depends strongly on isolation efficiency. As we show, optimal control policies can yield mitigation policies with similar infection rates to total shutdown but lower socioeconomic costs. However, optimal control policies can be fragile given mis-specification of parameters or mis-estimation of the current disease state. Hence, we leverage insights from the optimal control solutions and propose a feedback control approach based on monitoring of the epidemic state. We utilize genetic algorithms to identify a ‘switching’ policy such that susceptible individuals (both PCR and serological test negative) return to work after lockdowns insofar as recovered fraction is much higher than the circulating infected prevalence. This feedback control policy exhibits similar performance results to optimal control, but with greater robustness to uncertainty. Overall, our analysis shows that test-driven improvements in isolation efficiency of infectious individuals can inform disease-dependent interaction policies that mitigate transmission while enhancing the return of individuals to pre-pandemic economic activity.

## Introduction

As of 13 August 2020, more than 20,439,000 cases of coronavirus disease 2019 (COVID-19) have been reported worldwide with more than 744,000 deaths globally^1^. Starting at the reported origin of the pandemic in Wuhan, China, control measures have been implemented in most countries where outbreaks have occurred^2,3,4,5,6,7^. Multiple public health strategies are being deployed to slow outbreaks, and although recommendations always include social distancing and isolation of confirmed cases, the full spectrum of measures and levels of adherence differ from country to country, making assessments of strategy efficacy difficult (see^2^, controversy surrounding^8^)

The non-pharmaceutical control strategies for COVID-19 largely follow those employed in previous viral epidemics, including SARS, Ebola and MERS. Initial strategies can be broadly grouped into mitigation and suppression, where the former attempts to preserve essential health care services and contain morbidity and mortality, whereas the latter imposes more severe, emergency restrictions to prevent health care system collapse and provide conditions for easing-off towards less intense mitigation strategies^9^. Both mitigation and suppression approaches carry considerable social and economic costs, meaning that policymakers and the public at large only adopt them for short time periods^10^. A problem is that control measures have often been applied irrespective of an individual’s disease status (and/or likely infection risk severity) and are driven, in part, by the absence of information-driven alternatives.

Hence, distinct from lockdowns, there is an increasing interest in implementing population-wide prevention methods that decrease transmission risk while enabling economic re-engagement. Examples of such measures include mask-wearing^11,12^, contact-free interactions^13^, and restructuring of physical spaces^14^. The use of masks, in particular, has been shown to be effective at reducing respiratory transmission of SARS-CoV-2, particularly when individuals in a potentially infectious interaction are wearing a mask^15,16^. These population-wide measures still carry uncertainty since individuals are expected to behave uniformly irrespective of their disease status. As the scale of COVID-19 testing has increased, jurisdictions may also have an opportunity to consider implementing tactical mitigation strategies informed by testing.

Testing for infected status can, in theory, be used to initiate targeted isolation, identification and tracing of contacts, quarantining of contacts, and then selected testing of contacts^17^. If done rapidly and at scale, this kind of targeted PCR-based testing can provide early detection of cases and help break new chains of transmission^18,19^. The effectiveness of contact-tracing based control strategies hinges on the accurate identification and isolation of exposed and infectious cases. Slow return of test results (primarily) and false negatives (as a secondary factor) limit the effectiveness of test-based control policy^20^. A complementary tactic is the strategic deployment of recovered individuals identified by serological tests in infection hot-spots to effectively dilute transmission events^21,22^. Similar to the identification of infected individuals through PCR tests, immunity to SARS-CoV-2 is assessed through serological testing for protective antibodies^23,24^. Population-wide interventions, testing efforts, the selective confinement or deployment of people contingent on their infection status, and inaccuracies and limited adherence to policies, combine to create a challenging landscape for the persistent control of outbreaks^25^.

Non-pharmaceutical COVID-19 control until effective vaccines become available will necessarily involve periods of reduced social and economic activity; i.e., ‘business, but not as usual’. Control efforts are already generating hardship and could in the longer-term result in social unrest and increased mortality^26,27,28^. Here we confront a joint problem: how to identify policies that aim to reduce fatalities arising from COVID-19 while also enabling economic engagement. Such a joint goal necessarily has a tension, can lead to dichotomous ‘extremal’ policies, and requires confrontation through careful modeling. We do so in two stages. First, we use optimal control to assess both health and economic outcomes in an SEIR disease model framework. There is a substantial and growing literature on optimal control for COVID-19, the bulk of which focuses on non-personalized release policies or policies that target age- or risk-stratified groups^2,29,30,6,31,9^. Here, we identify optimal control policies to modulate interaction rates based on disease status identified by PCR and serological testing - unifying prior efforts centered on isolation and shield immunity. We find that intermediate policy outcomes can do nearly as well as strict public health scenarios, without incurring the severe costs as suppression-centered policies. However, optimal controls can be fragile, when applied in practice given that they rely on time- rather than state-based interventions; the consequence of mistiming interventions can be severe^30^, Hence, guided by the optimal control analysis, we identify state-dependent policies similar to feedback control that provide actionable guidance for individual behavior. As we show, using population-wide PCR and serological testing as the basis for mitigation (rather than surveillance) has the potential to yield dual benefits if carried out at population scales: reducing COVID-19 transmission while enabling more individuals to return to work sooner and with fewer restrictions than would otherwise be possible.

## Results

### Optimal control framework for state-dependent contact rates policies that balance public health and socioeconomic costs

**Figure 1:**
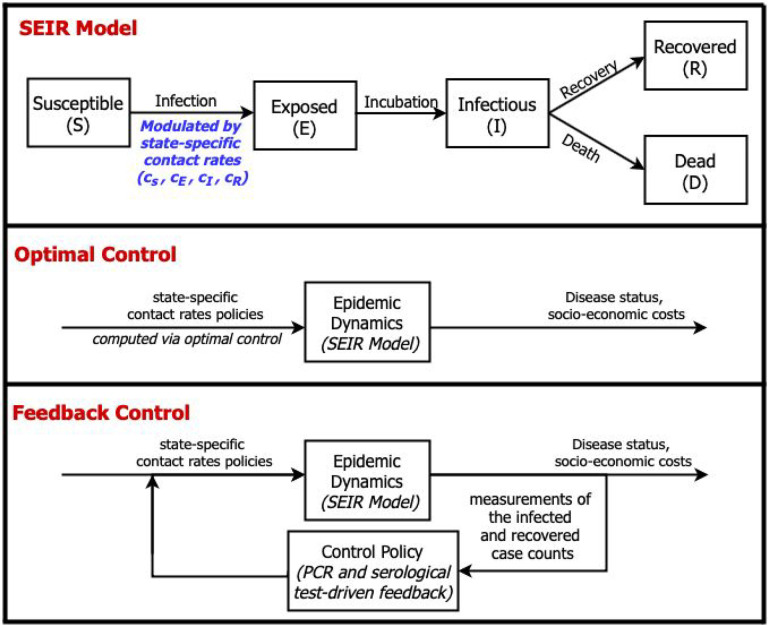
Epidemic dynamics with optimal and feedback control of disease-status driven contact rates. (Top) SEIR model schematic in which the force of infection is modulated by state-specific contact rates, see text and SI for details. (Middle) Diagram of optimal control approach: contact rates are pre-specified given model structure and estimate of parameters and current conditions. (Bottom) Diagram of feedback control approach: contact rates are updated in real-time based on measurements of the infected and recovered case counts via testing surveillance.

We develop an optimal control framework to identify policies that address the tension between decreasing contacts (that reduce new infections) with increasing contacts (that are linked to socio-economic benefits). We represent the epidemic using a Susceptible-Exposed-Infectious-Recovered (SEIR) nonlinear dynamic model (see Supplementary Information/Methods for complete details; see Figure 1). In doing so, the force of infection is influenced by state-specific contact rates *c*_*S*_ *, c*_*E*_ *, c*_*I*_ and *c*_*R*_ for susceptible, exposed, infectious and recovered individuals, respectively – these different levels form the basis for a control policy that directs individuals to interact at different levels depending on their test status.

In the optimal control framework, a set of state-specific contact rates are identified that minimize the appropriately weighted sum of what we term ‘public health’ and ‘socioeconomic’ costs. Public health costs are quantified both by average infected levels and cumulative deaths. Socioeconomic costs are quantified in terms of reductions in the total rate of interactions and by shifts in state-specific contact rates. The optimal control ‘solution’ is then a time-dependent set of disease-specific rates which are both shaped by and shape the epidemic itself (see SI/Methods for details on the gradient projection algorithm used to identify the solution). Note that we constrain the contact rate of exposed individuals to be equal to that of susceptible individuals given the challenges of timely identification of exposed individuals who are not yet infectious (and presumably have insufficient viral titer to be identified using screening tests; an issue we return to in the Discussion).

**Figure 2:**
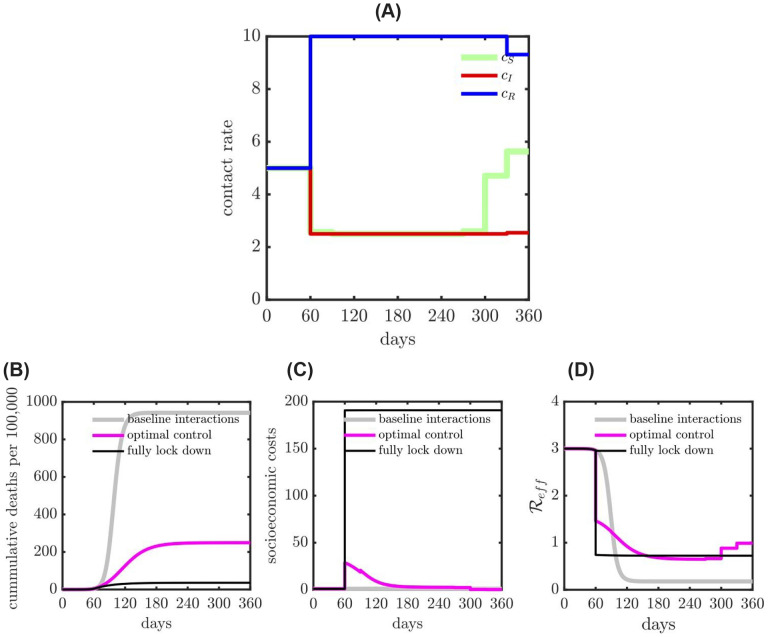
Comparison of health and economic outcomes of COVID-19 given various interventions: baseline interactions (i.e., no intervention); optimal contact rate intervention (balance both health and economic outcomes) and fully lock down intervention (applied to all the subpopulations) with 75% isolation efficiency. (A) The optimal contact rate with 50% isolation effectiveness and shield immunity level 2. (B) Cumulative deaths (health outcome) during the epidemic. (C) Socio-economic costs (economic outcome) during the epidemic. (D) Measure of effective reproduction number ($R_{eff}$) for different interventions during the epidemic.

Figure 2 shows the results of comparing a baseline outbreak (i.e., neglecting public health costs, given weighting parameter ξ=0) to a full lockdown scenario (i.e., neglecting socioeconomic costs with 75% isolation for all, ξ>>1) and a balanced scenario with optimized contact-rates (i.e., corresponding to ξ = 1). As shown in Figure 2, in the baseline scenario, the disease spreads through the population leading to 94% cumulative infection (as expected given strength-size relationships for $R_{0}=3$). In contrast, a full lockdown scenario with 75% reduction in contact rates of all individuals after 60 days leads to a total outbreak size of 4% of the population. The optimal control solution in the balanced case ξ = 1 reveals a potential route to jointly address public health and socioeconomic cost. From the perspective of public health, the optimal control solution leads to 25% cumulative infections. In addition, the socioeconomic costs in the optimal control case are higher in the short-term but approach that of the baseline scenario in the long-term. Indeed, the effective reproduction number identified via an optimal control framework in the balanced scenario gradually reduces to sub-critical levels (close to an effective reproduction number, $R_{eff}=0.75$) while gradually relaxing controls over time. The optimal control solutions are shown in Figure 2A. The optimal control solutions differ based on disease status, recovered individuals elevate their interactions, infectious individuals isolate, and susceptible individuals lock-down before gradually returning to pre-lockdown levels.

### Personalized, test-based optimal control policies and their impact on public health and socioeconomic outcomes.

**Figure 3:**
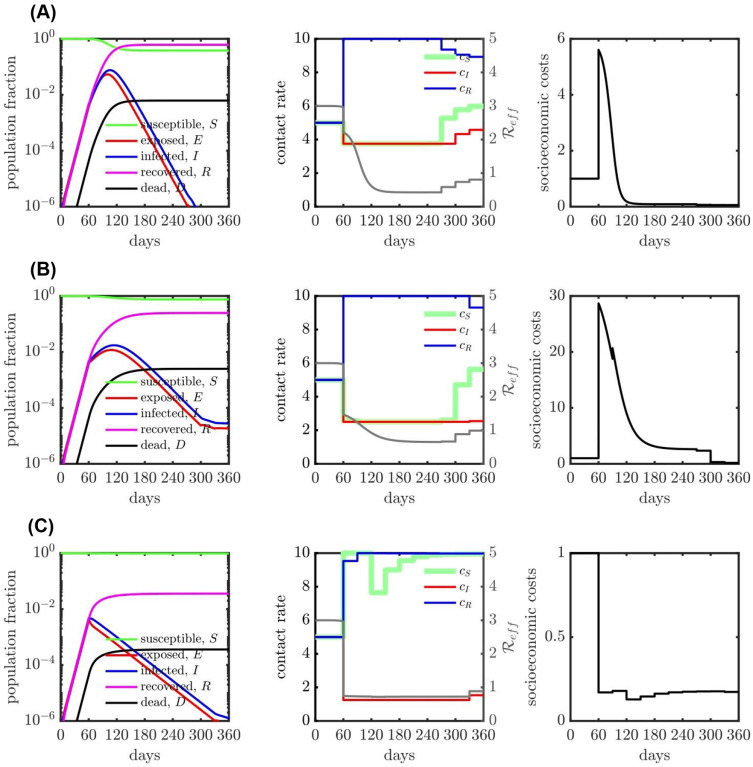
SEIR dynamics with contact rate interventions for various isolation efficiencies, (A) 25% isolation efficiency; (B) 50% isolation efficiency and (C) 75% isolation efficiency. The relative importance (ξ) is 1 for all the cases (A), (B) and (C). The contact rate interventions start at 60 days, people follow baseline (or normal) interactions before that. For all the isolation efficiency scenarios (three rows), the left panel shows the population dynamics given the optimal contact rate shown in the middle panel. The gray curve in the middle panel represents the measure of corresponding effective reproduction number ($R_{eff}$). The right panel shows the corresponding socio-economic costs. See SI/Methods for additional scenarios.

In order to explore the mechanisms identified by the optimal control framework, we systematically modulate the effectiveness of isolation and evaluate its effect on the state-dependent optimal contact rates and disease dynamics. In practice, isolation effectiveness is influenced by availability, accuracy, and speed of testing as well as fundamental limitations on an individual’s ability to isolate (which can vary with socioeconomic and other factors). Figures 3A–C evaluate low, medium, and high efficiency of isolation spanning 25%, 50%, and 75% reduction in the contact rates of infected individuals, respectively. As is evident, the optimal control solutions for state-dependent contact rates vary significantly with isolation effectiveness; suggesting that COVID-19 response policies that can vary with disease status may open up new possibilities to balance public health and socioeconomic outcomes.

First, in the low (25%) or medium (50%) effectiveness cases, susceptible, exposed, and infectious individuals adopt the maximal level of isolation. Inefficient isolation of infectious individuals elevates risks of new transmission that are not outweighed by socioeconomic benefits. Notably, the optimal control solution includes an *elevated* level of interaction by recovered individuals. This finding recapitulates ‘shield immunity’^21,22^, insofar as recovered individuals are protected from re-infection over the course of the intervention. The elevated contacts of recovered individuals have multiple effects: both diluting interactions by susceptibles (and reducing transmission risk) and by increasing socioeconomic activity. In contrast, for sufficiently high levels of isolation efficiency (75%), the optimal control solutions suggest there is no need for a general lockdown. Instead, the combination of infected case isolation and shielding by the subpopulation of recovered individuals is sufficient to rapidly reduce and contain $R_{eff}$below 1, leading to a decreasing number of new infections. We note that irrespective of isolation effectiveness, balancing public health and economic outcomes drives $R_{eff}$ below 1, but not necessarily to 0 (albeit, given the constraints imposed by lockdown efficiency, such an extreme reduction may not even be possible), and eventually increased immunity permits an easing-off in restrictions yielding an increase in $R_{eff}$^32^.

### Sensitivity of optimal control approach to mis-timed implementation of policies

Despite its potential to balance public health and socioeconomic costs, a central drawback of optimal control solutions is the potential exponential growth of errors. Given the fact the COVID-19 dynamics are only partially observed (with significant uncertainty in the actual state), application of a policy requires an estimate of the time since epidemic initiation, what we term ‘epidemic age’. In order to evaluate the sensitivity of optimal control policies due to mis-timing, we first computed the optimal control policy for a system one month after an outbreak. However, instead of implementing the policy matched to the actual epidemic age, we enforce the optimal control policy 30 days later, i.e. at the end of 60 days after the start of the outbreak. Figure S7 shows the difference in the mis-timed control policy vs. the optimal control policy; as is evident the mistimed policy relaxes stringent lockdown when the optimal policy continues to lock-down. As a consequence the total deaths are far higher for 25% and 50% isolation efficiency (see Table 1). The mistimed policy, in effect, biases the system towards minimizing socioeconomic rather than public health costs. This significant difference in performance metrics demonstrates the potential shortcomings of implementing a policy based on optimal control. However we note that with a stringent isolation efficiency, delays are less problematic. The reason is that with efficient infected case isolation, both the mis-timed and optimal control policy could enable nearly all individuals to work, given that $R_{eff}$ is held below 1 by infection isolation on its own.

Despite its fragility, we identify common features of the optimal control policy given variation in the effectiveness of infectious case isolation. First, the optimal control policy minimizes infected contact rates. The optimal control solutions also robustly identify an immune shielding strategy for recovered individuals, i.e., such that recovered individuals elevate their interactions to the maximum possible relative to baseline. Importantly, differences in the optimal control policy are primarily centered on identifying a switch point in contact rate level for the susceptible population. From Figures S4, S5 and S6, we observe the switch point as a function of time, showing that irrespective of isolated case effectiveness and shield immunity constraints, the increase in susceptible contact rates happens later in the lockdown period. Switchover points correspond to times when the infection prevalence is relatively low compared to the recovered population. This observation provides the basis for a feedback, rather than optimal, control policy.

### Feedback-control policy for balancing public health and socioeconomic costs

We propose the use of a feedback control policy adapted from emergent features of the optimal control policy solutions: (i) infectious individuals isolate as far as is possible; (ii) recovered individuals increase their activities as much as possible, i.e., akin to shield immunity. Hence, we set out to identify a system-dependent change in the contact rate of susceptible individuals, separating lockdowns vs. return-to-work. In practice, we identify a critical curve in *I-R* plane (i.e., infected-recovered cases plane) via a genetic algorithm, such that the recommended behavior of susceptible individuals is dictated by surveillance-based estimates of infectious and recovered individuals (see Supplementary Information for more details).

**Figure 4:**
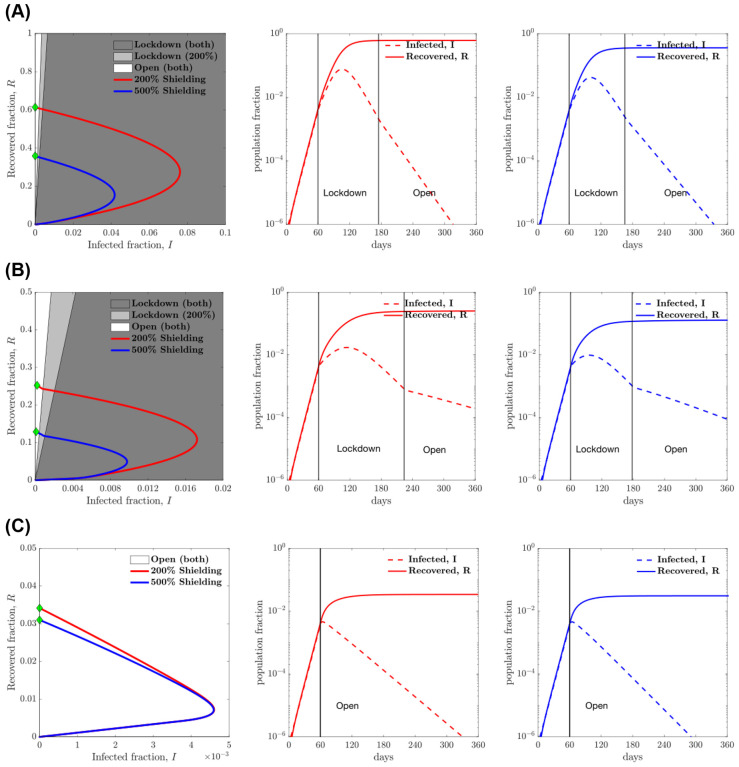
Heuristic state feedback intervention policies varying with isolation efficiency: (A) 25% isolation efficiency; (B) 50% isolation efficiency and (C) 75% isolation efficiency. The shielding levels considered here are 2 and 5 times the base contact rate (i.e., 200% shielding and 500% shielding respectively). An optimal line divides the plane into two regions which determines the optimal contact rate for the susceptible population for the current infected and recovered cases. The optimal policy in the dark grey region is lockdown for both shielding levels while the system with a higher level of shielding (level of 5) is open in the light grey region. The phase plots (red and blue curves) show the evolution of the infected and recovered case fractions over the period of 360 days, while applying the control strategy described above for shielding levels of 2 and 5 respectively. See SI/Methods for a larger set of plots with increments of 5% in isolation efficiency and for shielding levels of 2, 3, 4 and 5. The second and third plots show the infected and recovered cases for shielding levels of 2 and 5 respectively as functions of time. The vertical lines mark the time instances at which the policy is implemented with lockdown imposed and also the time at which it is lifted. For the case of isolation efficiency of 75%, no lockdown is needed at all for the susceptible population.

Figure 4 summarizes the results of the feedback control policy. From a policy perspective, for both 25% and 50% isolation effectiveness the feedback control policy identifies a switch between lock-down and return-to-work when there are significantly more recovered individuals than infectious individuals (approximately 100x as many). Hence, circulating case levels should be relatively low relative to recovered individuals before shifting from lockdowns to re-openings. We also examined the same scenarios while assuming recovered individuals return to work but without the beneficial effects of shield immunity. As is apparent, the policies are more stringent, with the critical relationship shifting to requiring that there are more than 350 times as many recovered individuals as infected individuals.

Critically, the performance of the test-driven feedback policy is nearly identical for the performance metrics with or without mis-timing (see Table 2). This finding implies that state-based approaches will be less likely to have exponentially mis-timed applications, and reinforces the need for population-scale testing for both active infections (via PCR) and prior infections (via serological and/or antigenic based testing that evaluates protective immune responses). We note that a simple policy with only two states - ‘lockdown’ and ‘open’, respectively corresponding to minimum and baseline contact rates for the susceptible cases, would be easier to implement than one with continuous ‘phases’ or state changes. In Figure S9, we document the generalizability of results given variation in infected case isolation and the level of shield immunity.

### Concluding remarks

We have developed a linked series of optimal- and feedback-control analyses to evaluate the effectiveness (and benefits) of modifying contact rates for managing the COVID19 pandemic from both health and economic perspectives. Throughout, our central goal was to optimize the interactions between individuals (in different compartments) so as to achieve a defined balance between public health and economic outcomes. By explicitly incorporating contact rates as the control variables in a SEIR model, we were able to identify optimal control policies that could, in theory, significantly reduce expected infections (and fatalities) while reducing the negative socioeconomic costs of sustained lockdowns. Optimal control policies are unlikely to be applied in practice, given the potential exponential mis-specification of policies over time. Hence, we leveraged insights from the optimal control solutions to guide a feedback control approach that performs nearly as well as the optimal control approach with significant improvements in robustness given uncertainty in estimating the epidemic state. Collectively, our control policies indicate that infected individuals should be isolated (as effectively as possible), recovered individuals should be encouraged to return to work (given benefits accrued via shield immunity), while the release of other individuals from lock-down should be guided by the epidemic state. The transition from lock-down to return-to-work occurs when circulating case loads are far lower than recovered case counts; with the scope of the epidemic sharply controlled by infection case isolation. A combination of policies, e.g., mask-wearing, physical distancing, will help to reduce transmission risk for individuals who do return to work.

We recognize that the SEIR framework is intentionally simplified. The epidemic model does not account for process or observational noise, analytic test features, heterogeneity, stratified risk, asymptomatic cases, and detailed elaboration of severe cases. By reducing the model complexity we have tried to shed light on the general problem of balancing public health with socioeconomic outcomes. In doing so, we have highlighted a middle ground between dichotomous outcomes that focus on public health or socioeconomic costs solely. Central to our work is the presumption that testing is sufficient to estimate both the circulating case counts and the number of recovered individuals. In doing so, serological testing is key. Given under-testing, the calibration of reported cases requires either model-based inference or serological test comparisons to estimate the ascertainment bias^33^. Collectively, we remain closer to the beginning than the end of the Covid-19 pandemic. As we have shown, accelerating the slow-down of transmission while restoring economic activity may be enabled by both personalized, test-driven, policies.

## Supplementary Material

1

## Figures and Tables

**Table 1:** Comparison between optimal control approach for contact policy with and without delay. The comparisons are made for isolation efficiencies of 25%, 50% and 75%, with performance metrics of total deaths (per 100,000 individuals) and working fraction. The total death is significantly higher for the system with delay when the isolation efficiency is not 75%, suggesting poor robustness to delay.

Optimal Control Approach	Without Delay		With Delay	
Efficiency of Isolation (shielding = 2)	Total Deaths (per 100,000 individuals)	Working Fraction	Total Deaths (per 100,000 individuals)	Working Fraction
25%	600	90.95%	720	94.96%
50%	250	67.13%	510	83.65%
75%	40	99.92%	40	99.95%

**Table 2:** Comparison between feedback control approach for contact policy with and without delay. The comparisons are made for isolation efficiencies of 25%, 50% and 75%, with performance metrics of total deaths and working fraction. Both performance metrics are nearly identical for all efficiencies, suggesting no significant effect of delay on the system.

Feedback Control Approach	Without Delay		With Delay	
Efficiency of Isolation (shielding = 2)	Total Deaths (per 100,000 individuals)	Working Fraction	Total Deaths (per 100,000 individuals)	Working Fraction
25%	620	89.98%	620	89.94%
50%	250	62.40%	250	62.12%
75%	30	99.90%	30	99.90%
